# Exposure to revised drinking guidelines and ‘COM-B’ determinants of behaviour change: descriptive analysis of a monthly cross-sectional survey in England

**DOI:** 10.1186/s12889-018-5129-y

**Published:** 2018-02-14

**Authors:** Abigail K. Stevely, Penny Buykx, Jamie Brown, Emma Beard, Susan Michie, Petra S. Meier, John Holmes

**Affiliations:** 1 School of Health and Related Research, University of Sheffield, Sheffield, UK; 2 Sheffield Alcohol Research Group, School of Health and Related Research (ScHARR), University of Sheffield, Sheffield, UK; 3 UK Centre for Tobacco and Alcohol Studies (UKCTAS), Nottingham, UK; 4 Department of Behavioural Science and Health, University College London, London, UK; 5 Department of Clinical, Educational and Health Psychology, University College London, London, UK

**Keywords:** Drinking guidelines, Health promotion, Evaluation, Alcohol consumption, Trend analysis

## Abstract

**Background:**

January 2016 saw the publication of proposed revisions to the UK’s lower risk drinking guidelines but no sustained promotional activity. This paper aims to explore the impact of publishing guidelines without sustained promotional activity on reported guideline exposure and determinants of behaviour (capability, opportunity and motivation) proposed by the COM-B model.

**Methods:**

Data were collected by a monthly repeat cross-sectional survey of adults (18+) resident in England over 15 months between November 2015 and January 2017 from a total of 16,779 drinkers, as part of the Alcohol Toolkit Study. Trends and associated 95% confidence intervals were described in the proportion of reported exposure to guidelines in the past month and measures of the capability, opportunity and motivation to consume alcohol within drinking guidelines.

**Results:**

There was a rise in reported exposure to drinking guidelines in January 2016 (57.6–80.6%) which did not reoccur in January 2017. Following the increase in January 2016, reported exposure reduced slowly but remained significantly higher than in December 2015. In February 2016, there was an increase in measures of capability (31.1% reported tracking units of alcohol consumption and 87.8% considered it easier to drink safely) and opportunity (84.0% perceived their lifestyle as conducive to drinking within guidelines). This change was not maintained in subsequent months. Other measures showed marginal changes between January and February 2016 with no evidence of change in subsequent months.

**Conclusions:**

Following the publication of revised drinking guideline in January 2016, there was a transient increase in exposure to guidelines, and capability and opportunity to drink within the guidelines that diminished over time. The transience and size of the changes indicate that behaviour change is unlikely. Well-designed, theory-based promotional campaigns may be required for drinking guidelines to be an effective public health intervention.

**Electronic supplementary material:**

The online version of this article (10.1186/s12889-018-5129-y) contains supplementary material, which is available to authorized users.

## Background

### Drinking guidelines and behaviour change

In 2012, as part of the UK Government’s Alcohol Strategy, the country’s Chief Medical Officers were asked to oversee a review of their lower risk drinking guidelines. The review aimed to “support individuals to make informed choices about healthier and responsible drinking” (p. 4). Key milestones in the process of updating the guidelines are summarised in Table 1. Two expert groups considered whether the guidelines should be updated and were persuaded by new high quality evidence, particularly relating to reduced certainty about cardio-protective effects from moderate drinking and increased certainty about risk of cancer. January 2016 saw the publication of proposed revisions to the drinking guidelines (DGs) which recommended that “To keep health risks from alcohol to a low level it is safest not to drink more than 14 units a week on a regular basis. If you regularly drink as much as 14 units per week, it is best to spread your drinking evenly over 3 or more days”.^1^ These were the first revisions since the previous guidelines were published in 1995. The weekly consumption guidance for men was reduced by approximately a third and the focus shifted from daily to weekly limits [1]. In the UK, a unit is 8 g of pure ethanol and so the guideline figure of 14 units per week is equivalent to 112 g of ethanol [2]. It is common for guidelines to provide separate recommendations for men and women, with weekly guidelines ranging from 60 to 190 g of ethanol for women and 70–288 g for men [3–5]. The Guidelines Development Group considered the evidence on the risk of alcohol-related harm faced by men and women and concluded that a single low risk guideline figure was justified because risks were similar at moderate consumption levels [2].

**Table 1:** Key milestones in updating UK lower risk drinking guidelines (2012–2016)

Milestone	Date
Review of lower risk drinking guidelines announced	March 2012
Expert group assessment of the need to update the guidelines	2013–2014
Guidelines development group considered the evidence and produced advice on revisions to the guidelines	2014–2015
Publication of proposed revisions	January 2016
Public consultation on the clarity of the guidelines concluded	April 2016
Final guidelines released	August 2016

After publishing the proposed revisions in January, the new guidelines were subject to a public consultation until April 2016 which focused on whether the recommendations and reasoning behind them were clear and easy to understand [2, 6]. The final guidelines were released in August 2016 after only minor changes to wording. Although there was minimal UK Government publicity in August, publication of the draft guidelines in January 2016 was accompanied by press releases and other prominent media activity from not only the UK Government, but also a range of other interest groups.

Guidelines, and their promotion, are prominent features of public health policy in the UK and beyond with governments or medical authorities publishing drinking guidelines in at least 37 countries [3]. However, international evidence for their effectiveness in reducing alcohol consumption is limited. There are a lack of high quality evaluations of the effects of promotional efforts and a literature review concluded that very little research has been conducted into their use for primary prevention [7]; although the available evidence generally indicates a lack of impact [8]. One limitation of previous work in this area is that it does not explore different stages or components of behaviour change from receipt of the information through to an actual shift in behaviour, and so the areas in which DG publication and promotion fails are not clear in the context of well-established theoretical frameworks. A detailed understanding of potential impacts of DGs on the determinants of behaviour change is important in order to consider methods to improve effectiveness.

### Behaviour change model

There are a number of theoretical frameworks available to understand processes of behaviour change and the model used was chosen as it integrates core constructs present in many earlier theories [9]. The model specifies that capability, opportunity and motivation interact as a system to generate behaviour (‘COM-B’ model). Capability is considered as being physical or psychological, opportunity as environmental or social, and motivation as automatic or reflective. In the context of drinking within guidelines, examples of capability would include knowledge of safe drinking, skills to track units and self-efficacy to drink within guidelines; opportunity would include suitability of lifestyle and access to information about cutting down; and motivation would include attempts, intention and desire to drink within guidelines. Prior evidence suggests that the publication and promotion of drinking guidelines can produce improvements in knowledge, as well as that guideline interventions are effective in changing reflective motivation [9]. However, evidence of effects of publishing or promoting drinking guidelines on other components of the COM-B model is lacking [10–12].

### Application of the COM-B model to drinking guidelines

This research aims to explore whether drinkers were exposed to the revised guidelines following their publication and promotion in January 2016 and whether capability, opportunity and motivation to drink within the revised guidelines changed.

## Methods

### Data

Data for this study were taken from the Alcohol Toolkit Study (ATS) which is a monthly repeat cross-sectional survey of approximately 1600 adults living in England. Data collected between November 2015 and January 2017 were included to provide a total sample size of 25,443. This timeframe includes two survey waves prior to the revised guideline publication and 13 subsequent waves. Data collection for January 2016 occurred in the week following publication of the proposed DGs. Since the Alcohol Toolkit Study was expanded for the purpose of evaluating the new low impact DGs in November 2015, data collection prior to this was not possible.

The ATS uses a type of random location sampling in which 171,356 ‘output areas’, each of which holds around 300 households, are stratified based on geographic area and socioeconomic status. Sampled strata are then randomly allocated to interviewers who choose the houses to visit and conduct computer-assisted interviews with one member of each household. This process continues until quota targets, based on factors which influence the probability of being at home, are met. Data are then weighted using an iterative sequence of weighting adjustments to match nationally representative profiles based on age, sex, social-grade, region, working status and children in the household. The sampling method used is often considered to be superior to standard quota sampling as the choice of properties approached is significantly reduced. As interviewers choose houses within their allocated strata, there is no definite gross sample size and response/refusal rates cannot be meaningfully calculated. The full ATS methods are described in the study protocol [13].

The first question of the AUDIT (Alcohol Use Disorders Identification Test) screening tool [14] was used to assess the drinking status of respondents. All survey participants were asked ‘How often do you have a drink containing alcohol?’ and those who responded ‘Never’ were classified as non-drinkers and excluded from analyses (9177 participants).

### Measures

#### Drinking guideline exposure

Respondents who reported that they were drinkers were asked: “Before this interview, have you ever heard of there being a recommended maximum number of alcohol units people should drink in a day or a week? This is sometimes known as a ‘drinking guideline’.” The response options were “Yes” or “No”. Those responding “No” were classified as ‘unexposed’ to the DGs while those responding “Yes” were asked: “In which of the following places, if any, have you seen, read or heard this drinking guideline figure mentioned in the last month?”. There were 12 possible responses to this question including the option “Have not seen it mentioned in any of these places”. Those responding that they had not seen it were also classified as ‘unexposed’ and those providing one or more place(s) that they had seen the guideline were classified as ‘exposed’.

#### COM-B determinants of behaviour

The main outcome measures were 10 questions with ordinal response scales designed to capture the capability, opportunity and motivation components of the COM-B model (Table 2). The design of these questions was influenced by questions which were found to effectively predict smoking behaviour in the Smoking Toolkit Study (STS) [15].

**Table 2:** Survey questions and response options

COM-B dimension	Response option	Total missing, ‘don’t know’ or ‘refused’
Capability		
Knowledge		
Item 1: What do you think is the most number of units you can personally drink in a day on a regular basis before it does significant harm to your health?	1^a^ – 7+ units	1358 (8.1%)
Perceived capability		
Item 2: How easy or difficult do you personally find it to drink three or fewer units of alcohol a day?	1. Extremely difficult – 7. Extremely easy^a^	141 (0.8%)
Skills		
Item 3: How often, if at all, do you keep track of how many units of alcohol you personally drink each week?	1. Never – 7. Always^a^	91 (0.5%)
Opportunity		
Social opportunity		
Item 4: How easy or difficult do you think your lifestyle makes it for you to personally drink three or fewer units of alcohol a day?	1. Extremely difficult – 7. Extremely easy^a^	184 (1.1%)
Item 5: Do you know where to go if you wanted advice or information on how to cut down on your drinking of alcoholic drinks?	1. I have no idea – 5. Yes, definitely^a^	112 (0.7%)
Motivation		
Reflective motivation		
Item 7: To what extent are you actively trying to avoid drinking more alcohol than is good for you?	1. Not at all – 5. Definitely^a^	116 (0.7%)
Item 9: To what extent do you intend to keep your drinking within safe limits?	1. Not at all – 5. Definitely^a^	110 (0.7%)
Automatic motivation		
Item 6: To what extent do you want to avoid drinking more than is good for you rather than just thinking that you should?	1. Not at all – 5. Definitely^a^	234 (1.4%)
Item 8: To what extent do you want to keep your drinking within safe limits?	1. Not at all – 5. Definitely^a^	108 (0.6%)
Item 10: Nowadays how concerned, if at all, are you about drinking more units of alcohol than is good for you?	1. Not at all concerned – 5. Definitely concerned^a^	94 (0.6%)

Key: ^a^The direction of the survey scale which was considered positive

Responses to these questions have been dichotomised into ‘positive’ and ‘negative’ groups as shown in Table 2 with either two (for five item scales) or three (for seven item scales) response categories falling into the positive group. The exception to this is Item 1 (Table 2), which asks how many units of alcohol can be regularly consumed in a day without significantly harming health, where the first two responses (1 unit, 2 units) have been selected as positive as this is consistent with the new lower risk DGs.

#### Analysis

Changes in responses to 10 COM-B measures over the 15 monthly datasets between November 2015 and January 2017 were examined using the 95% confidence intervals around the overall proportion of positive responses. December 2015 was used as a reference point to identify trends, since the draft guidelines were published in January 2016. All analyses were based on weighted survey data. Cases were excluded pairwise where the response to the question being analysed was ‘Don’t know’, ‘Refused’ or missing. The number of exclusions was highest for the first question, which related to knowledge of how many units it is safe to regularly consume (*n* = 1443). The number excluded for the rest of the measures was between 243 and 191. The same approach was used to assess change in reported DG exposure for which 2131 participants were excluded due to missing data on their exposure status.

To explore whether the method of dichotomising the COM-B measures affected results, sensitivity analyses were undertaken where five-point scales contained only one positive result (instead of two) and seven point scales contained only two positive responses (instead of three). A further sensitivity analysis was conducted for item 1, in which participants who responded ‘Don’t know’ were included in the negative response group. All analyses were conducted in IBM SPSS Statistics version 23.

## Results

### Drinking guideline exposure

**Fig. 1:**
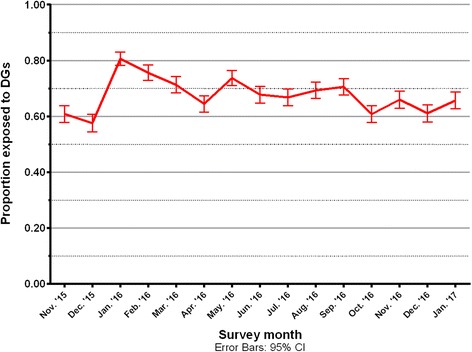
Trends in reported drinking guideline exposure

Drinkers’ self-reported exposure to DGs in any location rose from 57.7% in December 2015 to 80.6% in January 2016 (see Fig. 1). Figure 1 shows the trend in reported exposure with 95% confidence intervals. Subsequent to January 2016 this has gradually decreased but remained higher than in December 2015. Monthly trends, and associated 95% confidence intervals, are available for all measures (see Additional file 1).

### Responses to capability measures

**Fig. 2:**
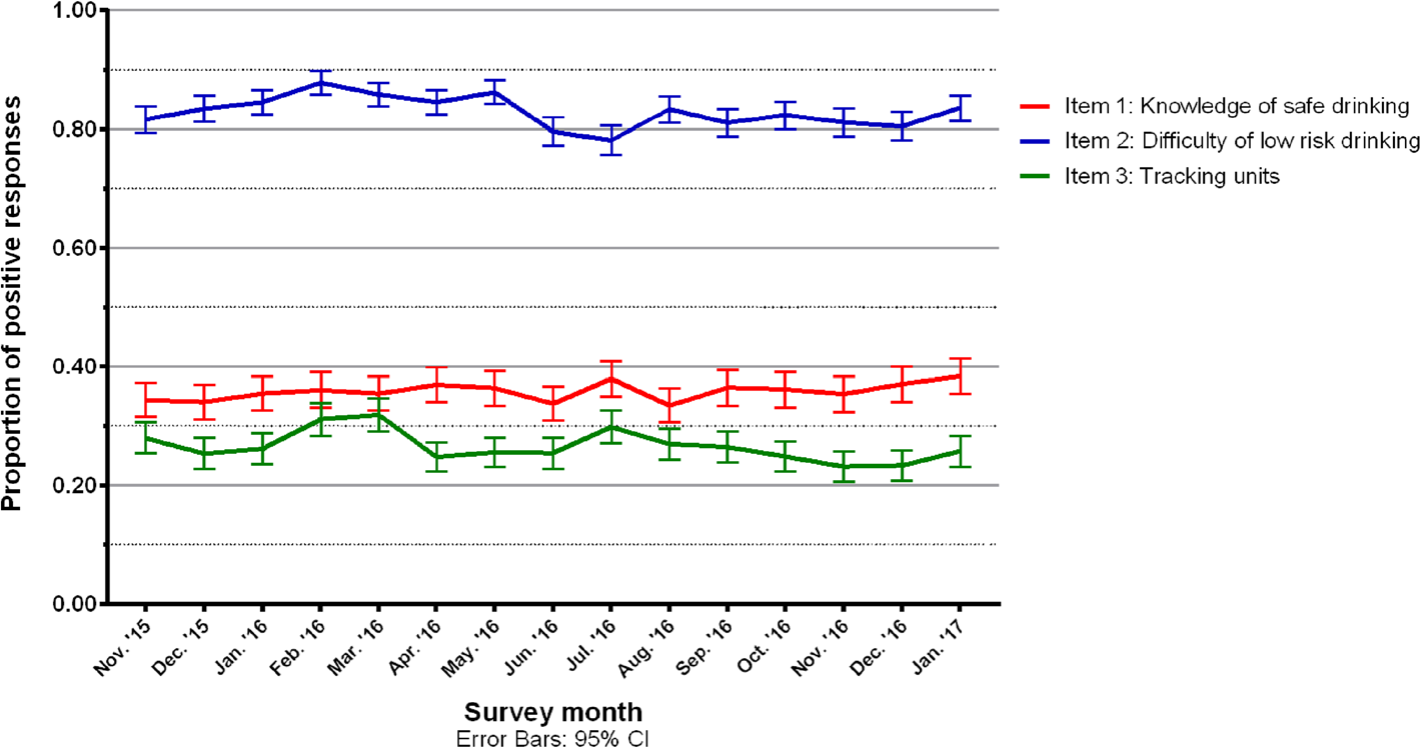
Trends in positive responses to capability measures

Among drinkers, 34.0% (31.5–37.2%) said that it was safe to regularly drink two or fewer units in December 2015. There was no clear trend in this proportion over time. The proportion of people who believed it was easy to drink less than three units a day and who tracked their units increased in February 2016 when compared with December 2015, although these improvements were modest (4.4 and 5.8%) and not maintained in the subsequent months (see Fig. 2).

### Responses to opportunity measures

In order to measure social opportunity factors, participants were asked how difficult their lifestyle makes it to drink three or fewer units per day. Responses to this measure also improved from December 2015 to February 2016: the proportion who reported that their lifestyle made it easy to drink three or fewer units per day rose from 77.9% (75.5–80.4%) to 84.0% (81.8–86.2%) but the improvement was also not maintained.

**Fig. 3:**
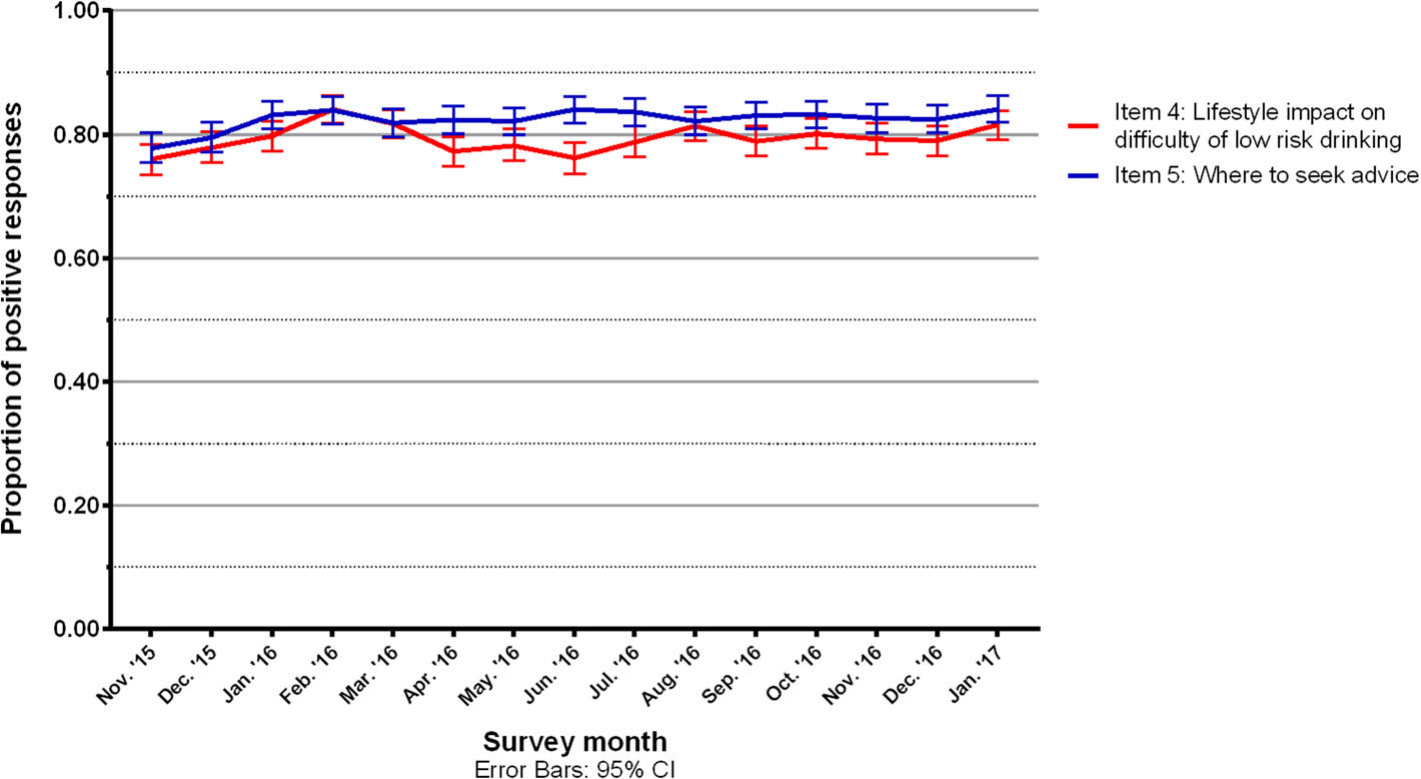
Trends in positive responses to opportunity measures

The second opportunity measure asked whether the respondent knew where to find information on cutting down their consumption of alcohol. There was no clear trend over time in positive responses to this (see Fig. 3).

### Responses to motivation measures

The first four questions designed to measure motivation (six, seven, eight and nine) focus on different aspects of motivation. The first two ask about drinking more than is good for you, whether the respondent ‘want/s to’ avoid it and whether they’re ‘actively’ trying to avoid it. The next pair relate to respondents keeping their drinking within safe limits and respectively ask whether they ‘want to’ or ‘intend to’ do so. The questions which ask about whether participants ‘want to’ engage in the specified behaviour are designed to assess automatic motivation while their counterparts assess reflective motivation.

The proportions of respondents who reported that they wanted to, or were actively trying to avoid drinking more than is good for them in December 2015 were lower (52.2 and 42.5%) than the proportions of people who wanted to, or intended to, keep their drinking within safe limits (77.0 and 77.0%). Trends in positive responses to these four measures were marginal between December and February.

**Fig. 4:**
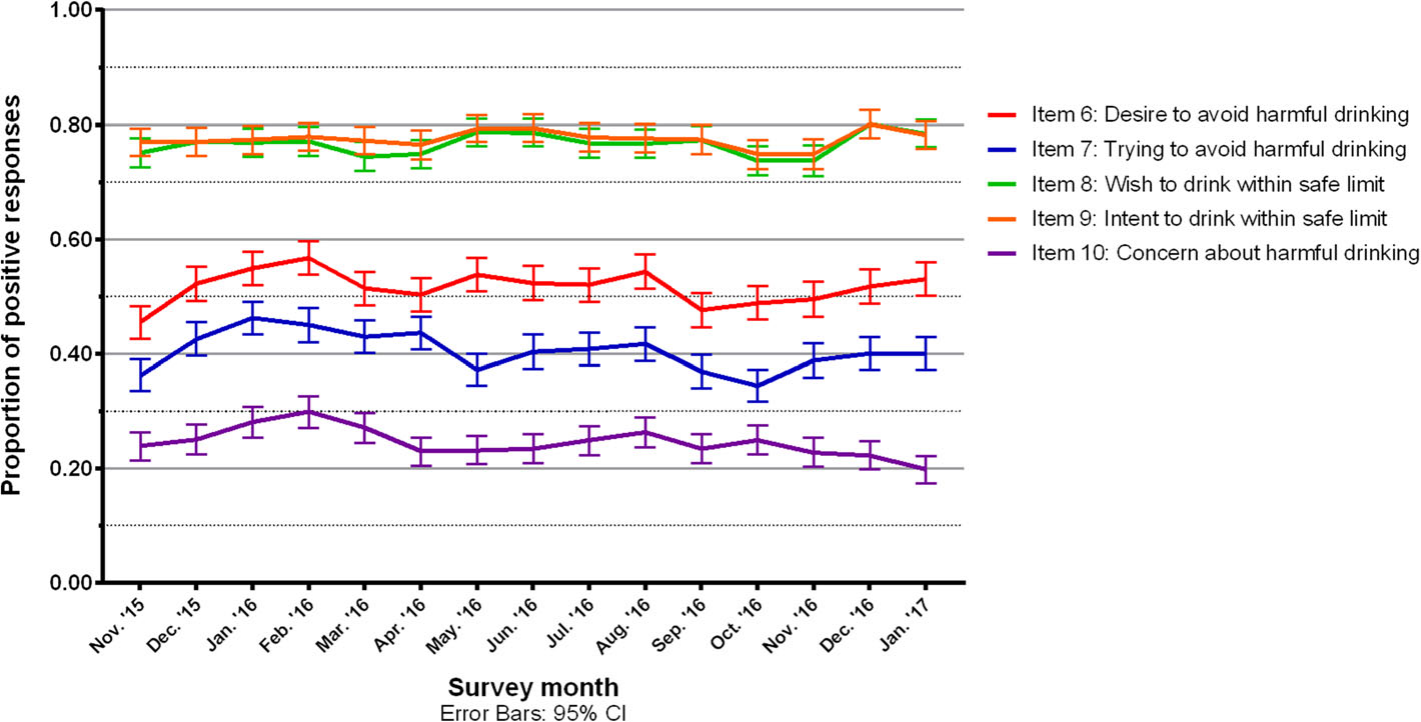
Trends in positive responses to motivation measures

Lastly, automatic motivation was further assessed by question ten, which asked how concerned each respondent was about drinking more than is good for them. In December 2015 this proportion was 25.0% (22.4–27.6%). There was no clear change in the proportion of respondents who were concerned over time (see Fig. 4). Sensitivity analyses for each of the ten COM-B measures did not significantly change the results.

## Discussion

We found that there was an increase in reported exposure to drinking guidelines in January 2016, which did not reoccur in January 2017. Following this, reported exposure fell, although remaining significantly higher than in December 2015. Following this rise in exposure, we found increases in measures of capability (proportion who reported tracking units of alcohol consumption and considered it easier to drink safely) and opportunity (proportion who perceived their lifestyle as conducive to drinking within guidelines). However, the change did not persist over subsequent months. Additionally, we observed some marginal changes in other measures.

A key strength of this research is its use of a theory-based framework for studying behaviour change (COM-B) in order to explore the short term effects of promoting lower risk DG [9]. Further strengths include those of the Alcohol Toolkit Study, which collects a nationally-representative sample of drinkers living in private households in England using consistent methods on a monthly basis which enabled analysis of pre- and post- intervention trends across multiple data points [13]. The relative robustness of the methods compared to previous studies and the theoretically informed approach are likely to strengthen the generalisability of the results to culturally-similar high income countries.

Among the key limitations are the potential confounding effects of December and January being traditionally heavy and light drinking months. However, data were collected over 15 months which facilitated comparison of results at guideline publication to those from the following year. Data collection was by self-report which is subject to known biases in studies on alcohol consumption; even though this study is not measuring consumption directly, issues such as social desirability may still have impacted responses [16, 17]. Since the results presented aggregate the responses of all participants, conclusions regarding the effect of publication of the new lower risk DGs on population sub-groups cannot be made. The question wording did not explicitly refer to the UK official government guidelines but we are unaware of other advice being widely interpreted as guidelines. Lastly, the proportion of non-drinkers in the ATS sample appears somewhat higher than other surveys in England: for example, the Adult Psychiatric Morbidity Survey (APMS) – which is the only other survey in England to also include the AUDIT – reported that 23% of respondents did not drink at all, while the equivalent figure for the ATS in 2014 was 29% [18]. Unlike the APMS, however, the ATS does not include a clarifying question and likely misclassifies a proportion of people who drink very rarely as ‘never’ [19].

Although previous evaluation of the efficacy of drinking guidelines to change behaviour is limited, this result is consistent with findings of low impact on those measures which have been explored [7, 8]. A contributing factor in this study may be the lack of large-scale organised promotion of the new low-risk DGs. For example, there have been no national mass media campaigns following the initial announcement of the guidelines and, despite over 80% of UK alcoholic product labels including the DGs [20], these labels have not been updated to give the new recommendations with guidance on how to do so only being published in March 2017 [21]. Additionally, reviews of the effectiveness of behaviour change efforts have consistently shown that achieving exposure is not sufficient to achieve effectiveness where interventions and campaigns are poorly designed. Promotion of drinking guidelines should therefore be designed with reference to prior theory and evidence on effective communication of messages and techniques for changing behaviours [22, 23]. This could be supported by the finding of this research that although reported exposure to drinking guidelines increased in January 2016 and remained above the level observed in December 2015, sustained change in the theoretical mediators of behaviour change was not demonstrated.

There was no difference in respondents’ knowledge of the number of units per day which it is safe to drink regularly over time (see Additional file 1). This could be seen as inconsistent with existing literature, which suggests that DGs can improve public knowledge of alcohol harms [10–12, 24]. Furthermore, the percentage of people who gave one or two units as the most units they could regularly drink on a single day before doing significant harm to their health was low in January 2016 (35.4%), meaning that most people thought that the level for low risk drinking was above that given in the new low-risk DGs. However, the measure of knowledge used here does not ask what the guideline figure is – rather it asks for the number of units that the respondent can regularly drink without significant health risk. It may be that this is interpreted as being different to the low-risk DG. Additionally, the number of units that it is safe to drink ‘regularly’ according to the low risk DGs is open to interpretation. It was not within the scope of this study to explore lay interpretations of the new guidelines or the COM-B measures used.

Given the results presented here, and the findings of low impact of DGs on alcohol consumption in the previous literature [7, 8], policy makers should consider the process of guideline implementation as well as additional or alternative methods to DGs when working to produce change in alcohol consumption. It is important to provide accurate information on the risks of alcohol consumption [4]. However, guidelines do not implement themselves; they require active, evidence-based strategies to support implementation [25, 26]. Furthermore, in order to build on the current findings, it is important to consider the impact of drinking guidelines on higher risk drinkers, who may view 14 units per week as unobtainable, and on health inequalities given the stark differences in risk faced by those in different socioeconomic groups [27–29].

## Conclusions

The publication and promotion of new low-risk drinking guidelines in January 2016 did not result in persistent improvements in UK adults’ capability, opportunity and motivation to reduce their alcohol consumption, despite an increase in reported drinking guideline exposure. Evaluations of well-designed, theory-based promotional campaigns are required to build the evidence about how to enable drinking guidelines to be an effective public health intervention.

## Additional file

Additional file 1: Trends in reported drinking guideline exposure and responses to COM-B measures. (DOCX 26 kb)

## Abbreviations

ATS: Alcohol Toolkit Study
AUDIT: Alcohol Use Disorders Identification Test
COM-B: Stands for: capability, opportunity, motivation and behaviour
DG: Drinking guideline
STS: Smoking Toolkit Study

## Footnotes

1 The previous guidelines (1995) recommended that men should not regularly consume more than 3–4 units a day and 2–3 units a day for women. The definition of regularly was every day or nearly every day.
