# Supplementary material for: Exposure to revised drinking guidelines and ‘COM-B’ determinants of behaviour change: descriptive analysis of a monthly cross-sectional survey in England
Source: BMC Public Health. 2018 Feb 14;18:251. doi: 10.1186/s12889-018-5129-y (PMC5813355; doi:10.1186/s12889-018-5129-y)
Supplement: Supplementary file 1 — Trends in reported drinking guideline exposure and responses to COM-B measures. (DOCX 26 kb) [file 12889_2018_5129_MOESM1_ESM.docx]

**Additional file 1. Trends in reported drinking guideline exposure and responses to COM-B measures.**

| Survey month | Exposure to drinking guidelines (95% CI) | Item 1: Knowledge of safe drinking  (95% CI) | Item 2: Difficulty of low risk drinking  (95% CI) | Item 3: Tracking units  (95% CI) | Item 4: Lifestyle impact on difficulty of low risk drinking  (95% CI) | Item 5: Where to seek advice  (95% CI) | Item 6: Desire to avoid harmful drinking  (95% CI) | Item 7: Trying to avoid harmful drinking  (95% CI) | Item 8: Wish to drink within safe limit  (95% CI) | Item 9: Intent to drink within safe limit  (95% CI) | Item 10: Concern about harmful drinking  (95% CI) |
| --- | --- | --- | --- | --- | --- | --- | --- | --- | --- | --- | --- |
| Nov. 2015 | 60.9%  (57.9-63.9) | 34.3%  (31.5-37.2) | 81.6%  (79.4-83.8) | 27.9%  (25.3-30.5) | 76.0%  (73.5-78.4) | 77.8%  (75.4-80.2) | 45.5%*  (42.6-48.3) | 36.2%*  (33.5-39.0) | 75.1%  (72.6-77.6) | 77.0%  (74.6-79.4) | 23.9%  (21.4-26.3) |
| Dec. 2015 | 57.6%  (54.5-60.8) | 34.0%  (31.0-36.9) | 83.4%  (81.2-85.6) | 25.3%  (22.7-27.9) | 77.9%  (75.5-80.4) | 79.5%  (77.1-81.9) | 52.2%  (49.2-55.2) | 42.5%  (39.6-45.5) | 77.0%  (74.5-79.5) | 77.0%  (74.5-79.5) | 25.0%  (22.4-27.6) |
| Jan. 2016 | 80.6%*  (78.2-83.1) | 35.4%  (32.6-38.3) | 84.5%  (82.4-86.6) | 26.1%  (23.5-28.7) | 79.8%  (77.4-82.1) | 83.1%  (80.9-85.3) | 54.9%  (52.0-57.8) | 46.2%  (43.3-49.1) | 76.9%  (74.4-79.4) | 77.4%  (74.9-79.8) | 28.1%  (25.4-30.7) |
| Feb. 2016 | 75.6%*  (72.9-78.4) | 36.0%  (33.0-39.0) | 87.8%*  (85.8-89.8) | 31.1%*  (28.3-33.8) | 84.0%*  (81.8-86.2) | 83.9%  (81.7-86.1) | 56.7%  (53.8-59.7) | 45.0%  (42.0-47.9) | 77.1%  (74.6-79.6) | 77.9%  (75.4-80.3) | 29.9%  (27.1-32.6) |
| Mar. 2016 | 71.3%*  (68.5-74.2) | 35.4%  (32.5-38.3) | 85.8%  (83.8-87.8) | 31.8%*  (29.1-34.6) | 81.7%  (79.5-84.0) | 81.8%  (79.6-84.1) | 51.4%  (48.5-54.3) | 42.9%  (40.1-45.8) | 74.4%  (71.9-77.0) | 77.2%  (74.8-79.7) | 27.1%  (24.5-29.7) |
| Apr. 2016 | 64.5%*  (61.5-67.4) | 36.9%  (34.0-39.8) | 84.5%  (82.4-86.6) | 24.7%  (22.2-27.2) | 77.3%  (74.9-79.7) | 82.3%  (80.1-84.6) | 50.3%  (47.4-53.2) | 43.6%  (40.7-46.4) | 74.9%  (72.4-77.4) | 76.5%  (74.0-79.0) | 23.0%  (20.5-25.4) |
| May. 2016 | 73.7%*  (71.0-76.4) | 36.3%  (33.4-39.1) | 86.2%  (84.2-88.2) | 25.5%  (23.0-28.0) | 78.2%  (75.8-80.9) | 82.1%  (79.9-84.3) | 53.78%  (50.9-56.7) | 37.2%  (34.4-40.0) | 78.7%  (76.3-81.0) | 79.3%  (77.0-81.7) | 23.1%  (20.7-25.6) |
| Jun. 2016 | 67.8%*  (64.8-70.8) | 33.7%  (30.8-36.6) | 79.5%  (77.1-81.9) | 25.4%  (22.8-28.0) | 76.2%  (73.6-78.7) | 84.0%  (81.8-86.1) | 52.3%  (49.3-55.3) | 40.3%  (37.4-43.3) | 78.6%  (76.2-81.0) | 79.4%  (77.0-81.8) | 23.4%  (20.9-25.9) |
| Jul. 2016 | 66.8%*  (63.8-69.8) | 37.9%  (34.9-40.9) | 78.1%*  (75.6-80.6) | 29.8%  (27.1-32.5) | 78.8%  (76.4-81.3) | 83.6%  (81.4-85.8) | 52.0%  (49.0-54.9) | 40.8%  (37.9-43.7) | 76.8%  (74.3-79.3) | 77.8%  (75.3-80.2) | 24.9%  (22.3-27.4) |
| Aug. 2016 | 69.3%*  (66.4-72.3) | 33.4%  (30.5-36.3) | 83.3%  (81.1-85.5) | 26.9%  (24.2-29.5) | 81.3%  (79.0-83.6) | 82.1%  (79.9-84.4) | 54.3%  (51.3-57.3) | 41.7%  (38.7-44.6) | 76.7%  (74.2-79.2) | 77.6%  (75.1-80.1) | 26.3%  (23.7-28.9) |
| Sep. 2016 | 70.6%*  (67.7-73.5) | 36.4%  (33.4-39.4) | 81.0%  (78.7-83.3) | 26.4%  (23.8-29.0) | 78.9%  (76.5-81.4) | 83.0%  (80.8-85.2) | 47.6%  (44.6-50.5) | 36.9%  (34.0-39.8) | 77.3%  (74.8-79.8) | 77.4%  (74.9-79.9) | 23.4%  (20.9-25.9) |
| Oct. 2016 | 60.8%  (57.8-63.9) | 36.1%  (33.1-39.0) | 82.3%  (80.0-84.5) | 24.8%  (22.3-27.4) | 80.1%  (77.8-82.5) | 83.2%  (81.0-85.4) | 48.8%  (45.9-51.8) | 34.4%*  (31.6-37.2) | 73.7%  (71.1-76.3) | 74.9%  (72.3-77.4) | 24.9%  (22.4-27.5) |
| Nov. 2016 | 66.0%*  (62.9-69.1) | 35.3%  (32.3-38.3) | 81.1%  (78.7-83.5) | 23.1%  (20.6-25.7) | 79.3%  (76.8-81.8) | 82.6%  (80.3-84.9) | 49.5%  (46.4-52.5) | 38.8%  (35.8-41.8) | 73.7%  (71.0-76.4) | 74.9%  (72.2-77.5) | 22.7%  (20.2-25.3) |
| Dec. 2016 | 61.1%  (58.0-64.1) | 37.0%  (34.0-40.0) | 80.4%  (78.1-82.8) | 23.3%  (20.8-25.8) | 79.0%  (76.6-81.4) | 82.4%  (80.2-84.7) | 51.7%  (48.7-54.7) | 40.0%  (37.1-42.9) | 80.1%  (77.7-82.5) | 80.1%  (77.7-82.5) | 22.2%  (19.8-24.7) |
| Jan. 2017 | 65.7%*  (62.8-68.7) | 38.3%  (35.4-41.3) | 83.6%  (81.4-85.7) | 25.7%  (23.1-28.2) | 81.5%  (79.2-83.8) | 84.0%  (81.9-86.2) | 53.0%  (50.1-55.9) | 40.0%  (37.2-42.9) | 78.4%  (76.0-80.8) | 78.2%  (75.8-80.6) | 19.8%*  (17.4-22.1) |
| * Significant differences compared to December 2015 based on 95% confidence intervals. | | | | | | | | | | | |
